# MoSGrid – a molecular simulation grid as a new tool in computational chemistry, biology and material science

**DOI:** 10.1186/1758-2946-3-S1-P14

**Published:** 2011-04-19

**Authors:** G Birkenheuer, D Blunk, S Breuers, A Brinkmann, I dos Santos Vieira, G Fels, S Gesing, R Grunzke, S Herres-Pawlis, O Kohlbacher, N Kruber, J Krüger, U Lang, L Packschies, R Müller-Pfefferkorn, P Schäfer, H-G Schmalz, T Steinke, K-D Warzecha, M Wewior

**Affiliations:** 1 University of Paderborn, 33098 Paderborn, Germany; 2 University of Cologne, 50923 Köln, Germany; 3 Technical University of Dortmund, 44221 Dortmund, Germany; 4 Eberhard-Karls-University of Tübingen, 72074 Tübingen, Germany; 5 Technical University of Dresden, 01187 Dresden, Germany; 6 Konrad-Zuse-Zentrum für Informationstechnik, 14195 Berlin, Germany

## 

The MoSGrid (Molecular Simulation Grid, http://www.mosgrid.de) project aims to provide remote computational chemistry services within the German Grid Initiative (D-Grid).

Submission and monitoring of compute jobs, as well as the retrieval of postprocessed results are realized through a web based portal. The use of standardized portlets and a generally modular approach allows for the simultaneous and independent implementation of frontends for different molecular simulation codes. To date, functional prototypes of portlets for applications from the quantum chemical and the molecular dynamics domain are available, being represented by Gaussian and Gromacs, respectively. The implementation of other quantum chemical codes, as requested by the community, and of codes for docking simulations is in preparation.

MoSGrid will furthermore foster efficient and collaborative work by providing secure but shareable repositories for validated data, as well as for reusable recipes and workflows [1-3].
